# The association of mitochondrial morphology and supercomplex redistribution with skeletal muscle oxidative capacity in older adults

**DOI:** 10.14814/phy2.70359

**Published:** 2025-05-07

**Authors:** Mauricio Castro Sepulveda, Sylviane Lagarrigue, Francesca Amati

**Affiliations:** ^1^ Aging and Muscle Metabolism Laboratory, Department of Biomedical Sciences University of Lausanne Lausanne Switzerland; ^2^ Service of Endocrinology, Diabetology, and Metabolism, Department of Medicine Lausanne University Hospital Lausanne Switzerland

**Keywords:** ATP_max_, electron transport chain, mitochondrial elongation, respirasome

## Abstract

Skeletal muscle maximal oxidative capacity (ATP_max_) is a key component of age‐related sarcopenia and muscle health. The contribution of mitochondrial morphology and electron transport chain supercomplex (SC) assemblies to ATP_max_ has yet to be determined in human muscle. ATP_max_ measured in vivo by ^31^phosphorus magnetic resonance spectroscopy in the *quadriceps femoris* of nine volunteers (65.5 ± 3.3 years old) was correlated with muscle biopsy outcomes before and after 4 months of supervised exercise. Mitochondrial morphology was assessed in electron micrographs, and SCs were measured by blue native gel electrophoresis. In the sedentary conditions, ATP_max_ was positively associated with complex (C) I and CIII in SC I+III_2_+IV_n_ and negatively associated with CI and CIII in SC I+III_2_. Regarding mitochondrial morphology, ATP_max_ was positively associated with markers of mitochondrial elongation. Exercise training‐induced increases in ATP_max_ were accompanied by mitochondrial elongation and by the redistribution of free complex III. Indicators of mitochondrial elongation were associated with the redistribution of specific complexes to SC I+III_2_+IV_n_. Higher skeletal muscle oxidative capacity in older adults is associated with mitochondrial elongation and the redistribution of electron transport chain complexes into higher rank SCs in the same muscle. Further, we provide evidence that mitochondrial elongation favors mitochondrial SC assembly.

## Graphical abstract

ATPmax is positively associated with mitochondrial elongation and the redistribution of electron transport chain complexes into higher‐order supercomplex assemblies in the skeletal muscle of elderly volunteers, both before and after a 4‐month supervised exercise intervention.

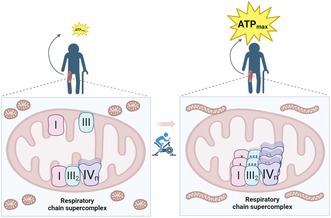

## 1 INTRODUCTION

In human skeletal muscle (SkM), mitochondrial oxidative capacity plays a crucial role in various health conditions and biological processes, such as type 2 diabetes and age‐related sarcopenia. The gold standard for evaluating mitochondrial oxidative capacity in vivo is the rate of phosphocreatine recovery post‐SkM contraction by ^31^phosphorus magnetic resonance spectroscopy (^31^P‐MRS), which reflects the maximal rate of oxidative ATP synthesis (ATP_max_) (Conley et al., 2000). In the context of aging, lower ATP_max_ levels have been linked with age (Gouspillou et al., 2014), decreased resting metabolic rate (Zampino et al., 2020), walking speed (Coen et al., 2013), muscle strength (Zane et al., 2017), susceptibility to fatigue (Santanasto et al., 2015), and predicted future decline in mobility among well‐functioning older adults (Tian et al., 2022).

The hypothesis of mitochondrial content being a primary determinant of mitochondrial oxidative capacity in human SkM is prominent. However, there is evidence of a mismatch between markers of mitochondrial content and ATP_max_. For example, in one of our exercise intervention studies, previously sedentary volunteers increased mitochondrial volume density on average by 50% with intervention, but their ATP_max_ only increased by 22% (Broskey et al., 2014).

Alongside mitochondrial content, mitochondrial morphology, dictated by the balance between mitochondrial fusion and fission, could regulate ATP_max_. Gemmink et al. (2023) highlighted that fused mitochondrial networks coincide with elevated mitochondrial respiratory capacity. We have shown a positive association between ATP_max_ and the content of Mitofusin 2, a mitochondrial pro‐fusion protein, and a negative association with dynamin‐related protein1 (DRP1), a pro‐fission protein (Arribat et al., 2019). To our knowledge, the relationship between mitochondrial morphology and ATP_max_ in human SkM remains unexplored.

Mitochondria transduce energy through oxidative phosphorylation using the electron transport chain (ETC). This system consists of four complexes (C), CI to CIV, that oxidize the reducing equivalents of NADH and FADH_2_ to create a proton gradient across the mitochondrial inner membrane. This gradient enables the production of ATP through complex CV, also known as ATP synthase. ETC respiratory complexes coexist alongside super‐assembled structures termed supercomplexes (SCs) (Lapuente‐Brun et al., 2013). SCs optimize metabolic flux and minimize ROS while ensuring efficient ATP synthesis (Guaras et al., 2016; Lapuente‐Brun et al., 2013). In our prior work, we observed that exercise training in previously sedentary older adults not only increased individual complexes but also prompted a specific redistribution of complexes into SCs (Greggio et al., 2017). Particularly, the relocation of CIII into SC I+III_2_+IV_n_, known as the respirasome, correlated with peak oxygen consumption (VO_2_peak) (Greggio et al., 2017). How much SC assemblies impact ATP_max_ is yet unknown.

The aim of this study was to explore the association between mitochondrial morphology, mitochondrial SC assembly, and ATP_max_ in SkM of older volunteers under sedentary conditions and after exercise training.

## 2 METHODS

### 2.1 Study design

This study integrates new data with previously utilized information, combined in a secondary analysis of nine volunteers who participated in the study “AGIR” for aging and insulin resistance. The data used previously were published in Greggio et al. (2017) and pertains to these nine volunteers whose muscle specimens were utilized to measure the redistribution of complexes in SCs as illustrated in figure 3 and detailed in table 2 in Greggio et al. (2017). ATP_max_, BMI, lean body mass (LBM), and exercise capacity were acquired and published in Arribat et al. (2019). Here, we present for the first time mitochondrial morphology measured using transmission electron microscopy (TEM) micrographs used previously for a different outcome (i.e., mitochondrial volume density measured by stereology) (Arribat et al., 2019).

**FIGURE 1:**
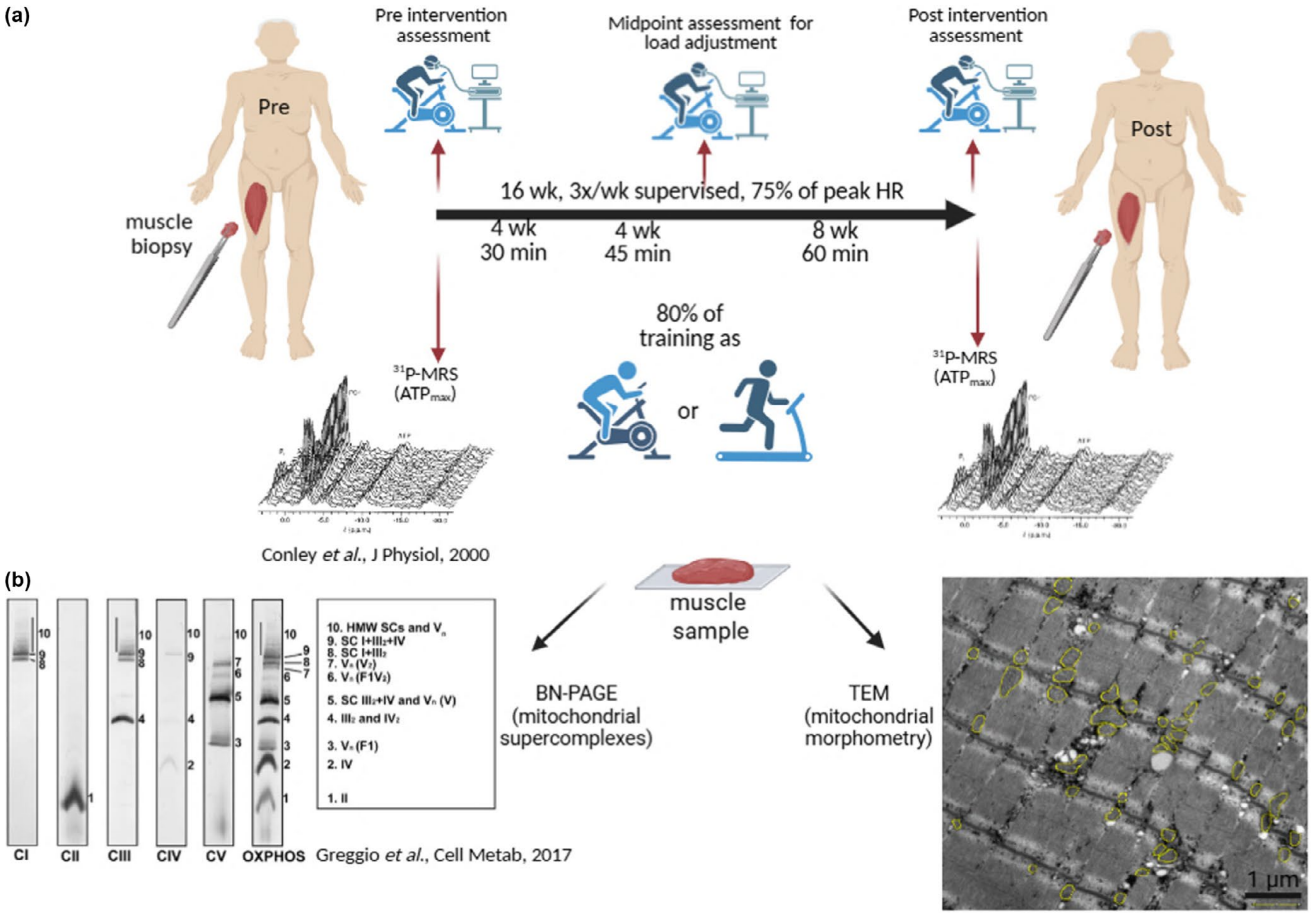
Study protocol. (a) Timeline, assessments, and intervention. (b) Methods used to measure electron transport chain supercomplexes and mitochondrial morphometry.

All outcomes were obtained before and after a 16‐week supervised exercise intervention detailed in Broskey et al. (2014). The study protocol is presented in Figure 1 and was approved by the Ethics Committee of the Canton of Vaud. All participants gave written informed consent.

### 2.2 Subjects' characteristics and in vivo ATP_max_

Participants were four females and five males, sedentary (<1 exercise session/week), nonsmokers, with a mean age of 65.5 ± 3.3(SD), mean BMI 28.1 ± 4.6 kg/m^2^, mean LBM 54.4 ± 10.1 kg, and mean VO_2_peak/LBM 38.8 ± 4.9 mL/kg/min.

ATP_max_ was computed as the product of the PCr recovery rate constant (k) and the resting PCr content measured by ^31^P‐MRS, assuming a constant ATP concentration of 8.2 mmol/L, as detailed in Broskey et al. (2014).

### 2.3 SkM biopsies and SCs quantification

Muscle biopsies were obtained after an overnight fast from the *vastus lateralis* under local anesthesia as previously described (Broskey et al., 2013). After trimming, 5 mg were fixed in glutaraldehyde for TEM, and 30 mg were immediately frozen in liquid nitrogen and stored at −80°C for SC quantification. SCs were measured using blue native gels as described in Greggio et al. (2017).

### 2.4 TEM and mitochondrial quantitative morphometry

TEM micrographs were obtained as previously described (Broskey et al., 2013). Mitochondrial morphology was evaluated in a blind manner using longitudinal sections. Each mitochondrion was drawn along the outer membrane. Partial mitochondria were not included. Once all mitochondria were circled, overlays were saved and processed using the automated particle analyses from Fiji (Schneider et al., 2012). Mitochondrial number is the average number of mitochondria per fiber. Size is the area of each mitochondrion. Perimeter measures the length of the circle drawn; Feret and minFeret diameters are maximum and minimum widths. Circularity is a roundness index where 1 is a perfect circle. Taken together, these outcomes allow obtaining a comprehensive quantitative assessment of mitochondrial morphology.

### 2.5 Statistics and computations

Relationships between variables were assessed with Spearman's rank‐order correlation. Percent changes (Δ) between pre‐ and post‐intervention data were calculated to assess the effects of the exercise intervention. Given the known relationship between ATP_max_ and age (Coen et al., 2013; Tian et al., 2022; Zampino et al., 2020; Zane et al., 2017), which tended to be confirmed in this cohort (*r* ^2^ = 0.37, *p* = 0.08), ΔATP_max_ was adjusted to explore the effects of the intervention independently of age using linear regression between exercise training‐induced ΔATP_max_ (dependent variable) and age (independent variable). Residuals ΔATP_max_ (measured ΔATP_max_ − expected ΔATP_max_) represent age‐adjusted ΔATP_max_. Prism 7 (GraphPad Software, La Jolla, CA) was used for all analyses.

## 3 RESULTS

**FIGURE 2:**
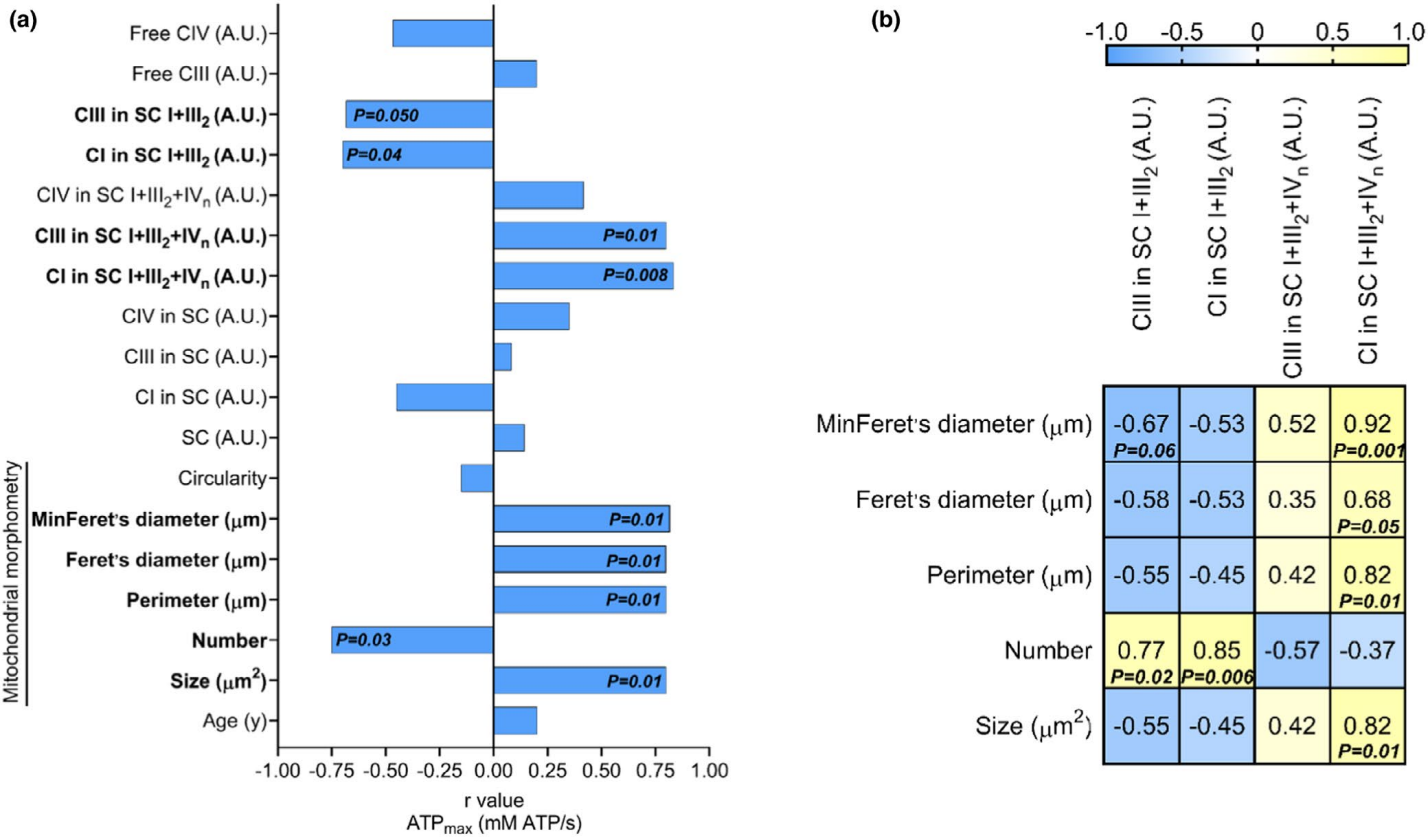
Higher ATP_max_ is associated with mitochondrial CI and CIII redistribution from SC I+III_2_ to SC I+III_2_+IV_n_ and an elongated mitochondrial phenotype in older adults SkM. (a) Spearman correlation (r) between ATP_max_ and multiple mitochondrial SC assemblies, morphometry parameters, and age. (b) Spearman correlation matrix (r and *P* value) between parameters of mitochondrial morphometry and SCs containing CI and CIII. *n* = 9.

In sedentary conditions, ATP_max_ was positively associated with the amount of mitochondria complex CI in SC I+III_2_+IV_n_ and CIII in SC I+III_2_+IV_n_ (Figure 2a). ATP_max_ was negatively associated with the amount of CI in SC I+III_2_ and CIII in SC I+III_2_ (Figure 2a). Regarding mitochondrial morphology, ATP_max_ was positively associated with size, perimeter, Feret, and MinFeret, but negatively associated with the number of mitochondria (Figure 2a).

Mitochondrial size, perimeter, Feret, and MinFeret were positively associated with the amount of CI in SC I+III_2_+IV_n_ (Figure 2b). Mitochondrial number was positively associated with the amount of CI and CIII in SC I+III_2_ (Figure 2b). MinFeret tended to be negatively associated with CIII in SC I+III_2_ (Figure 2b).

**FIGURE 3:**
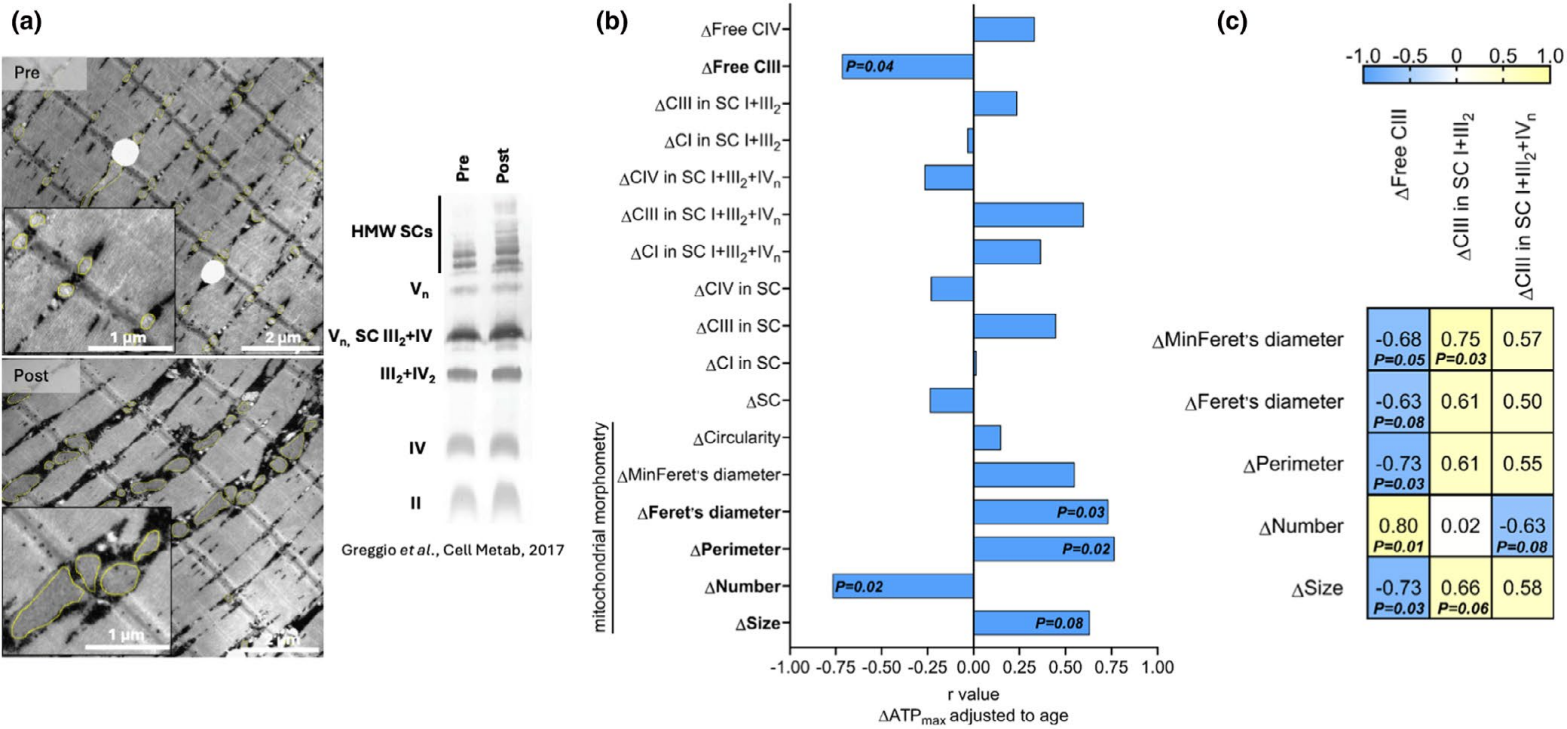
Exercise‐induced increase in ATP_max_ is related to exercise‐induced decrease in mitochondria free CIII and to increase in mitochondrial elongation in older adults SkM. (a) Representative electron micrographs and blue native gels from the same volunteer before and after the exercise intervention. HMW is high molecular weight. (b) Spearman correlation (*r*) between exercise‐induced changes in ATP_max_ adjusted to age with changes in multiple mitochondrial SC assemblies and mitochondrial morphometry parameters. (c) Spearman correlation matrix (*r* and *p* value) between exercise‐induced changes in parameters of mitochondrial morphometry and SCs containing CIII. All changes (Δ) are expressed in %. *n* = 9.

Representative electron micrographs and blue native gels from the same volunteer, before and after the exercise intervention, are shown in Figure 3a. Age‐adjusted ΔATP_max_ was negatively associated with Δfree CIII and Δnumber of mitochondria (Figure 3b), and positively associated with Δperimeter and ΔFeret (Figure 3b). Δsize and Δperimeter were negatively associated with Δfree CIII (Figure 3c). ΔMinFeret was positively associated with ΔCIII in SC I+III_2_; Δsize tended to show the same association (Figure 3c). Δnumber was positively associated with Δfree CIII and tended to have a negative association with ΔCIII in SC I+III_2_+IV_n_ (Figure 3c).

## 4 DISCUSSION

The molecular mechanisms that control ATP_max_ in older adults SkM are complex and not fully understood. Here, we demonstrate that in sedentary older individuals, elevated ATP_max_ is influenced by both the redistribution of CI and CIII from SC I+III_2_ to SC I+III_2_+IV_n_, and by an elongated mitochondrial phenotype. Increases in ATP_max_ after 4 months of exercise training are accompanied by the elongation of mitochondria and by the redistribution of free CIII to higher order SCs. Our findings suggest that the elongated mitochondrial phenotype, both in sedentary conditions and post exercise intervention, facilitates the redistribution of specific complexes to SC I+III_2_+IV_n_, thereby enhancing mitochondrial oxidative capacity.

The relationship between mitochondrial morphology and function has been studied over time to gain insight into the potential role of mitochondrial dynamics (Picard et al., 2013). Recently, Gemmink et al. (2023) observed that an elongated mitochondrial network was more compatible with higher maximal mitochondrial respiratory capacity (state U) than a fragmented network. Genetic disorders with imbalances of elongation/fragmentation are known to impact in vivo SKM oxidative capacity, such as in dominant optic atrophy due to mutations of the pro‐fusion gene *Opa1* (Lodi et al., 2011).

Previous studies in older volunteers implied SkM mitochondria pro‐fusion responses with exercise training interventions (Arribat et al., 2019; Axelrod et al., 2019) or changes in oxidative capacity measured in vivo (Broskey et al., 2014). To our knowledge, this is the first observation of an elongated phenotype post‐intervention associated with increases in ATP_max_ in the same muscle.

The positive association between ATP_max_ and the amount of CI or CIII in SC I+III_2_+IV_n_, combined with the negative association with CI or CIII in SC I+III_2_, suggest that the redistribution of CI and CIII toward the respirasome impacts mitochondrial oxidative capacity. This is in accord with the hypothesized functions of SCs, which are to organize and optimize the flux of electrons, control ROS production, and modulate the activity of ETC complexes (Cogliati et al., 2021).

As recently reviewed by Picard and Shirihai (2022), mitochondria morphological changes underlie intra‐mitochondria functional changes to optimize the coupling of oxygen consumption to ATP synthesis and best match the dynamic metabolic state. When focusing on the relationships between morphological outcomes and the redistribution of complexes into SCs in sedentary conditions, we observed a coherent pattern where mitochondrial size, perimeter, and both Feret diameters were positively associated with the amount of CI in the respirasome (Figure 2b). After intervention, specific morphological parameters point to the relationship between mitochondrial elongation and the amount of CIII in the respirasome (Figure 3c). These observations suggest a role of mitochondrial morphology, in sedentary conditions and after exercise training, in SC assembly. Potential mechanisms regulating the association between mitochondrial morphology and mitochondrial SC assembly may involve both OPA1 and DRP1. OPA1 has been shown to increase SC assembly (Cogliati et al., 2013), while DRP1 has been shown to decrease it (Tomkova et al., 2019). It is possible that mitochondrial elongation facilitates SC assembly via protein and/or phospholipid exchanges. Further studies are needed to explore these mechanisms.

This study is not without limitations. First, it is based on observational and correlational results. Secondly, despite the relatively small sample size (*n* = 9), we observed significant correlations between mitochondrial SC assembly, mitochondrial morphology, and in vivo ATP_max_. Post hoc analyses revealed that the effect size of the correlations ranged from |ρ| 0.909 to 0.978. Thirdly, we were not able to measure cristae density or morphology, which would have been important to support seminal mechanistic publications linking fusion and tighter cristae to SC assembly in cells overexpressing Opa1 (Cogliati et al., 2013).

The strength of this study lies in the integration of in vivo ATP_max_ measurement with detailed analyses of mitochondrial morphology and SCs. We demonstrate the correlation between ATP_max_ and mitochondrial elongation, as well as the redistribution of ETC complexes into higher order SC assemblies. These findings suggest the potential for manipulating mitochondrial elongation to enhance SkM oxidative capacity in conditions characterized by lower SkM oxidative capacity, such as sarcopenia and insulin resistance.

## 5 CONCLUSION

This study demonstrated that, in older adults SkM, higher ATP_max_ is associated with mitochondrial elongation and the redistribution of electron transport chain complexes into higher order SCs. These findings are consistent with the conclusion that a mitochondrial elongated phenotype favors mitochondrial SC assembly.

## AUTHOR CONTRIBUTIONS

All experiments were performed in the laboratory of FA. Conceptualization: MCS and FA; methodology: MCS, SL, and FA; investigation: MCS, SL, and FA; resources: FA; writing—original draft: MCS and FA; writing—review and editing: MCS, SL, and FA; funding acquisition: FA.

## FUNDING INFORMATION

This work was supported by the Swiss National Science Foundation (188789, 170062, 149398, and 126339).

## CONFLICT OF INTEREST STATEMENT

None to declare.

## ETHICS STATEMENT

This study was approved by the Ethics Committee of the Canton of Vaud and conducted in accordance with the Declaration of Helsinki. Informed consent was obtained from all participants, and their confidentiality was maintained throughout the study.

## Data Availability

The data that support the findings of this study are available on request from the corresponding author.
